# Evaluation of Liposomal Bupivacaine Use in Elective Cerebral Aneurysm Surgery

**DOI:** 10.1227/neuprac.0000000000000143

**Published:** 2025-05-15

**Authors:** Brandon Laing, Randall W. Treffy, Cayla Jannsen, Emily Morris, Gerard MacKinnon, Hirad S. Hedayat

**Affiliations:** Department of Neurosurgery, Medical College of Wisconsin, Milwaukee, Wisconsin, USA

**Keywords:** Cerebral aneurysm, Cost-effectiveness, Liposomal bupivacaine, Long-acting local anesthetic, Postoperative pain control

## Abstract

**BACKGROUND AND OBJECTIVES:**

Liposomal bupivacaine (LB) is a long-lasting formulation of local anesthetic which can be effective up to 72 hours postoperatively. Although it is approved for postsurgical analgesia through local wound infiltration and has frequently been used in spine surgery, there is little evidence regarding the effect of LB on cranial surgery postoperative care.

**METHODS:**

We retrospectively reviewed 79 patients who underwent elective craniotomy for unruptured anterior circulation aneurysms, of which 44 were given LB and 35 were not. Postoperative pain scores, length of stay (LOS), opioid use, and cost of pain medication were all obtained from the patients' charts and analyzed.

**RESULTS:**

There was no significant difference in initial pain scores, average pain scores, opioid use, or intensive care unit or overall LOS. However, the total cost of pain medication when taking into account the cost of LB was significantly higher in the LB group compared with the control group ($647.84 ± 122.50 compared with $284.77 ± 113.44; *P* < .0001).

**CONCLUSION:**

Our data illustrate that LB does not seem to affect average pain score, total opioid use, opioid cost, or LOS but does seem to be associated with an overall increase in total cost of pain medication owing to the significant cost of LB. We suspect that although pain control is important in cranial surgery, the effect of LB is not substantial enough to affect overall pain control in these patients and is not a primary driver of hospitalization. However, further prospective, randomized studies would be helpful to evaluate the overall benefit of LB on cranial surgery outcomes.

**ABBREVIATIONS:**
IV: intravenous
LB: liposomal bupivacaine
LOS: length of stay
MME: morphine milligram equivalents.

Liposomal bupivacaine (LB) is an extended-release local anesthetic which can achieve local anesthesia for up to 72 hours postoperatively.^1^ The pharmacokinetics of bupivacaine are somewhat unique as bupivacaine has a high protein-binding percentage (95%), allowing it to have an extended half-life of around 3.5 hours.^2^ Placement of bupivacaine in a liposomal formulation consisting of biodegradable cholesterol, phospholipids, and triglycerides allows the bupivacaine to be released slowly, thereby increasing the duration of release of bupivacaine up to an estimated 72 to 96 hours after administration.^2,3^

The historic role of LB in postoperative analgesia is relatively novel and limited thus far. LB was first approved for surgical site infiltration for hemorrhoidectomy and bunionectomy in October 2011. Previous studies have shown variable benefits of LB affecting length of stay (LOS) and perioperative opioid requirements in spinal surgery patients.^4,5^ Although there is evidence of the benefits of LB in spine surgery, there is a paucity of literature evaluating its efficacy in cranial surgery. In a previous study evaluating the benefits of LB in pediatric Chiari decompression surgery, the use of intraoperative LB led to decreased pain scores and opioid requirements in the first 24 hours postoperatively.^6^ Given the lack of literature regarding LB's role in cranial surgery, the objective of this study was to assess its utility in elective anterior circulation aneurysm surgery.

## METHODS

### Patient Selection

Our study consists of a retrospective cohort review of 79 patients who underwent elective clipping of unruptured anterior circulation cerebral aneurysms at Froedtert Memorial Lutheran Hospital by the senior author between October 2020 and February 2024. The study was approved by the Medical College of Wisconsin Institutional Review Board. Patients were included if they had elective cerebral aneurysm surgery for an unruptured anterior circulation aneurysm. Patients who had ruptured aneurysms or were left intubated postoperatively were excluded as they would not fully benefit from the use of LB, given their diminished neurological status. A few patients were excluded from the study because of other complex medical or social issues that required preoperative admission and/or prolonged hospitalization for reasons unrelated to their surgery. Patients were grouped based on whether they received intraoperative LB administered within the surgical wound during the procedure. All patients consented for the proposed procedure.

### Surgical Intervention and Postoperative Care

Every patient underwent either a pterional or lateral supraorbital craniotomy for their elective aneurysms clipping completed by the senior author (HH). If LB was used, it was administered at the end of the procedure. In brief, 20 mL of LB was injected throughout the temporalis muscle and fascial layer as well as the loose connective tissue layer above the muscular fascia, not specifically targeted toward the neurovascular bundle. All patients were admitted to the intensive care unit (ICU) postoperatively for close neuromonitoring. The postoperative pain regimen consisted of oral acetaminophen with supplemental opioid medication as needed. The standard oral treatment medication opioid prescribed was oxycodone, whereas the standard parenteral opioid used was fentanyl. Alternative medications included hydromorphone, hydrocodone-acetaminophen, and in 1 patient, buprenorphine. Alternative medications were used in cases where there was a lack of benefit with medication on postoperative pain or patient preference.

### Data Collection and Chart Review

Information regarding postoperative intravenous (IV) and oral narcotic use, dosage, and hospital and ICU LOS were obtained from retrospective chart review. Oral and IV narcotic dosages were converted into morphine milligram equivalents (MME) to achieve comparative equipoise between opioids. The conversion of various opioids to MME is presented in Table 1. Both total and average daily amounts of IV opioid, oral opioid, and total MME were evaluated. The average amounts of total, IV, and oral MME per day were calculated based on each individual patient's LOS.

**TABLE 1.** Conversion Table: MME Conversion Table Used for This Study

Medication (dose)	MME (mg)
Oral oxycodone (5 mg)	7.5
Oral hydromorphone (1 mg)	4
Oral tramadol (1 mg)	0.1
Oral hydrocodone (7.5 mg)	7.5
IV fentanyl (25 mcg)	3
IV hydromorphone (0.2 mg)	4

IV, intravenous; MME, morphine milligram equivalents.

Patient's documented pain scores were obtained from the chart. Pain scores were scaled from 1 to 10 with “1” being minimal to no pain and “10” being severe pain as obtained by nursing staff for clinical evaluation. Individual opioid pharmacological medication cost was obtained from Froedtert Hospital's financial department. Total pharmacological cost was determined using the standard cost of each dose of medication and route (ie, oral vs IV) and multiplied by the number of doses received.

Data were collected and stored in SPSS Statistical (International Business Machines Corporation) and Microsoft Excel (Microsoft Corporation), and data were analyzed using GraphPad Prism (GraphPad Software Inc). Mann-Whitney tests were performed comparing 2 numerical variables whereas the Fisher exact test was used to evaluate the significance of categorical variables. The Spearman Rank test was used to evaluate correlation between continuous variables. An alpha level *P* < .05 was used to determine significance. Data points are displayed with mean and 95% CI as determined in Microsoft Excel. Our study design and reporting here adheres to the Strengthening the Reporting of Observational Studies in Epidemiology (STROBE) guidelines.^7^

The study was approved by the Medical College of Wisconsin Institutional Review Board 7/31/2017; PRO00030059.

## RESULTS

### Population Opioid Use and LOS

Seventy-nine patients underwent elective clipping of anterior circulation aneurysms. Forty-four were given LB intraoperatively whereas 35 were not. The average age of the 2 cohorts was not significantly different (60.41 ± 4.68 years for the LB group compared with 59.43 ± 4.36 years; *P* = .5972) while 77.3% of the LB group were female and 91.4% of the control group were female (*P* = .1290). 47.7% of the patients given LB underwent a pterional craniotomy whereas 45.7% of the non-LB cohort underwent a pterional craniotomy (*P* = 1.000). The remaining patients from each group underwent a lateral supraorbital craniotomy. The mean total narcotic use for the cohort was 65.61 ± 14.84 MME. The average daily opioid use was 21.88 ± 4.49 MME per day. Average LOS was 3.38 ± 0.52 days. 24 patients did not require any postoperative IV opioids. Six patients did not require oral or IV opioids.

### LB and Opioid Requirements

Differences in total, oral, and IV MME between the LB and control groups were evaluated. Total opioid use was similar between the LB group compared with the control group (55.95 ± 16.29 MME compared with 77.76 ± 26.97 MME; *P* = .3046; Table 2). Total mean oral opioid requirements were also similar between the LB group compared with the control population (44.95 ± 14.96 MME compared to 66.07 ± 25.52 MME; *P* = .2331; Table 2). Total mean IV opioid use was also similar between the LB group and the control group (13.80 ± 8.52 MME compared with 11.69 ± 6.126 MME; *P* = .2857; Table 2). When compared using the Fischer exact test, patients who received LB (16/44; 36.4%) were just as likely as those who did not (8/35; 22.9%) to require IV pain medications (*P* = .2258) and were just as likely as not to require no narcotic pain medication (4/44; 9.1% in LB group; 2/35; 5.7% in no LB group) (*P* = .6882).

**TABLE 2.** Study Results: Differences Between the LB and Control Group

Variable	LB	Control	*P* value
Total MME	55.95 ± 16.29	77.76 ± 26.97	.3046
Oral MME	44.95 ± 14.96	66.07 ± 25.52	.2331
IV MME	13.80 ± 8.52	11.69 ± 6.26	.2857
Pain score	3.55 ± 0.641	4.05 ± 0.749	.1611
LOS (d)	3.16 ± 0.649	3.66 ± 0.881	.0778
Total pharmacologic cost	$647.84 ± 122.50	$284.77 ± 113.44	<.0001

IV, intravenous; LB, liposomal bupivacaine; LOS, length of stay; MME, morphine milligram equivalents.

There was no significant difference in MME between the 2 groups. There was also no difference in pain scores or LOS in days. However, there was a significant increase in cost associated with the use of LB. Data are shown with average ± 95% CI. *P* value is obtained from Mann-Whitney test.

### LB and Postoperative Pain Scores

There was no significant difference in initial preoperative pain scores between the LB group and the control group (2.11 ± 1.08 compared to 1.71 ± 1.11; *P* = .6433). The pain score, as documented by nursing, averaged over the hospitalization was not significantly different between the LB and control groups (3.55 ± 0.64 compared with 4.05 ± 0.75; *P* = .1611; Table 2). When focusing on the pain score average of the day of surgery after completion (postoperative day 0), there is no significant difference between the LB and control groups (4.10 ± 0.88 compared with 4.37 ± 0.96; *P* = .4290), which remains the same for the first day after surgery (postoperative day 1) (3.69 ± 0.62 compared with 4.20 ± 0.80; *P* = .1926). There is also no significant difference between the maximum pain score from the LB vs control group (7.09 ± 0.78 compared with 7.46 ± 0.89; *P* = .2515). Of note, in the LB group, 5 patients (11.4%) did not have a documented pain score on postoperative day 0 and were therefore excluded from the comparison. Every patient in the control group had documented pain scores for postoperative day 0.

For the LB group, the oral (rho = 0.744; 0.511-0.875, *P* < .0001), IV (rho = 0.385; 0.003 + 0.0670, *P* = .043), and total narcotic (rho = 0.784; 0.579-0.896, *P* < .0001) use were statistically and positively correlated with average pain score as was the case for the non-LB group (oral; rho = 0.452; 0.063-1.000, *P* = .026: IV; rho = 0.490; 0.031-0.778, *P* = .033: total; rho = 0.605; 0.194-0.835, *P* = .006). For both groups, preoperative pain score was not statistically significantly correlated with oral, IV, or total narcotics.

### LB and Hospital LOS

The average LOS for the entire cohort was 3.38 ± 0.52 days with an average ICU LOS of 1.53 ± 0.24 days. There was no significant difference in average LOS (3.16 ± 0.65 days compared with 3.66 days ± 0.88; *P* = .0778; Table 2) or average ICU LOS (1.48 ± 0.35 days compared with 1.60 ± 0.32 days; *P* = .3143) between the LB group and control group.

### LB and Postoperative Opioid Cost

The direct cost of narcotic medication was evaluated within the patient cohort. Total average total price for narcotics for the cohort was $283.60 ± 82.60. There was no significant difference between postoperative narcotic cost between the LB group and the control group ($282.68 ± 122.50 compared with $284.77 ± 113.44; *P* = .3212). However, when including the cost of the LB used, the LB group had a substantially and significantly higher total pain medication cost ($647.84 ± 122.50 compared with $284.77 ± 113.44; *P* < .0001; Table 2).

## DISCUSSION

This study analyzed the potential benefits of LB in patients undergoing elective craniotomy for clipping of anterior circulation aneurysms. Based on our results, LB does not seem to have a significant effect on LOS, pain scores, narcotic use, or narcotic cost and we saw an overall increase in total pain medication cost, given the substantial cost of LB itself. Our data also indicate a positive and statistically significant correlation between pain scores and oral, IV, and total narcotic use indicating that we have a fairly good correlation between self-reported pain scores and narcotic use. This also was in the setting of no correlation between preoperative pain scores and narcotic use, indicating narcotic use in our patients seems correlated with postoperative pain only.

Previous studies have illustrated the benefits of LB in reducing postoperative opioid requirements. In a meta-analysis evaluating the benefits of LB infiltration, Byrnes et al^8^ evaluated 12 clinical trials including a total of 2512 patients and found reduced LOS and morphine requirements when compared with standard analgesia. However, they noted that there was low confidence in these estimates and recommended that further trials be performed. In the spine population, LB infiltration has also shown benefits of decreased opioid use, improved pain scores, shorter hospital stays, and lower overall hospital costs.^9^ Although the literature supports the benefits of LB in other surgeries, this is the first study to our knowledge evaluating the benefits of LB in cranial surgery exclusively.

The effect of LB on LOS seems to be somewhat variable. In our cohort, there was no difference in average LOS between the LB group and controls. It is likely that postoperative analgesia has little effect on LOS in patients undergoing elective aneurysm surgery. In a retrospective analysis, the main dependent predictors of extended hospital stay in patients undergoing elective craniotomy were age >70 years, American Society of Anesthesiologists class 3, patient race and ethnicity, functional status, diabetes, and evidence of postoperative complications such as postoperative hemiplegia, urinary tract infection, or pulmonary embolus.^10^ Other studies have reported similar findings that postoperative complication and need for extended rehabilitation are the primary drivers of LOS in patients undergoing elective cranial surgery.^11^ Thus, it is likely that the main drivers of LOS in elective craniotomies is less related to postoperative analgesia and primarily postoperative neurological complications, potentially requiring further rehabilitation needs.

When looking at the effects of LB in the spine literature, the results continue to be variable depending on the type of procedure. In patients undergoing anterior cervical diskectomy and fusion, LB did not have any effect on LOS.^12^ Conversely, in patients undergoing lumbar spine fusion, there is evidence that LB may lead to decreased LOS.^9^ These findings suggest that the benefit of LB is specific to the procedure performed. We suspect that LB is more beneficial in patients undergoing extensive muscle dissection as the postoperative pain in these cases can likely lead to impaired mobility and further physical therapy requirements. This is less of an issue in cranial surgery because the muscle dissection is often relatively minimal and does not affect muscles that affect patient mobilization beyond chewing with temporalis involvement.

### Cost-Analysis

Our findings do not show a benefit in reduction of overall pharmacological cost between the LB and control groups; in fact, when adding in the cost of LB, we found a significant increase in total pharmacological cost of $363.07. This lack of benefit is likely due to the finding that LB did not reduce overall MME requirements and itself is quite costly.

Previous evidence regarding the cost-effectiveness of LB has been quite variably debated in the literature. In the spine literature, patients who had LB had lower LOS, and increased mobility postoperatively, but had an overall $218 higher pharmacological cost per patient compared with the controls.^9^ Conversely, LB administration has shown to lead to lower overall hospital cost savings in both the orthopedic and colorectal literature.^13,14^ Ultimately, similar to the effect of LB on LOS, the potential for cost-reduction of this medication is likely to vary based on type of surgery performed making definitive evaluation of its overall economic benefit difficult. As mentioned above, increased LOS (and therefore increased cost) is likely not driven by pain control in cranial surgery.

In discussing the benefits of LB in cranial surgery, it is important to evaluate previous efforts to improve management of postoperative analgesia in this population. In a randomized controlled trial by Yang et al,^15^ the authors evaluated the effect of local scalp nerve block in elective cerebral aneurysm surgery. In their study, they compared scalp blocks with local anesthetic of 0.75% ropivacaine and their overall effects on postoperative inflammatory response, hemodynamic response, and postoperative pain control in patients undergone elective surgical clipping. Ultimately, they found that scalp nerve blocks lead to decreased local inflammatory response cytokines and lower postoperative pain scores.

### Limitations

There are multiple limitations to this study. First, this is a retrospective cohort study at a single institution, limiting the generalizability of our results. Second, although all patients had elective anterior circulation aneurysm surgery, the timing and dose of intraoperative LB was not standardized. As previously mentioned, there are clearly variable results of LB regarding opioid medication use, LOS benefit, and cost based on the type of procedure performed. Thus, to accurately evaluate the benefits of LB in the elective aneurysm clipping population, a randomized-control trial would likely need to be performed. Further limitations come from our approach for pain measurement, which is determined by nursing assessment, and although numerical pain assessments are reliable,^16^ the numbers we obtained were not designed for a trial but instead designed for patient assessment. The amount of opioid pain medication and route is also not standardized in our postoperative patients, and different patients may have different access to IV and/or oral narcotics based on postoperative medication orders placed by the surgical team and covering clinical team. Third, although we were able to obtain information regarding postoperative opioid cost, we were unable to obtain information regarding the total hospital charges for the patients in our cohort. Thus, there may be alternative effects of LB on total hospital cost savings that we were unable to evaluate in our study.

## CONCLUSION

This is the first study to evaluate the efficacy of LB in supratentorial cranial surgery. Our findings demonstrate no significant difference between our 2 groups other than an increase in total pain medication cost owing to the price of LB itself. Our study is limited by its retrospective nature and lack of standardization and randomization of patients undergoing LB administration. Further prospective randomized studies would help better elucidate the clinical benefit and cost-effectiveness of LB in cranial neurosurgery.
